# Body mass index has consistent effects on the magnitude of gait variability while its effect on the temporal structure of gait variability is age-dependent

**DOI:** 10.1007/s00421-026-06307-5

**Published:** 2026-06-18

**Authors:** Narges Shakerian, Seung Kyeom Kim, Tyler M. Wiles, Nick Stergiou, Aaron D. Likens

**Affiliations:** 1 Division of Biomechanics and Research Development, Department of Biomechanics and Center for Research in Human Movement Variability, University of Nebraska at Omaha, Biomechanics Research Building, 6160 University Drive South, BRB 201, Omaha, NE 68182 USA; 2 Department of Physical Education and Sports Science, Aristotle University, Thessaloniki, Greece

**Keywords:** Aging, Walking, Locomotion, Biomechanics, Body weight

## Abstract

**Purpose:**

High body mass leads to changes in gait to accommodate the increased mechanical demands. Thus, previous research has examined how body mass affects gait variability (i.e., stride-to-stride fluctuations). However, these studies have been focused on the magnitude (extent of these fluctuations) of gait variability, while the effects of body mass on the temporal structure (how fluctuations are organized over time) remain unexplored. This study examined how body mass, quantified using body mass index (BMI), affects both magnitude and temporal structure of gait variability in young (24.66 ± 2.79 years), middle-aged (45.77 ± 5.85 years), and older adults (64.70 ± 7.65 years).

**Method:**

We analyzed stride interval and stride length of 117 participants in NONAN GaitPrint dataset. Magnitude of gait variability was quantified using standard deviation (SD) and coefficient of variation (CoV). Temporal structure was assessed by Hurst exponent (H), which characterizes gait variability correlation over time. Linear mixed models examined the effects of BMI, age, and their interaction.

**Results:**

Higher BMI significantly predicted greater magnitude of variability for both stride interval and stride length, independent of age. For the temporal structure, a significant interaction of BMI and age was observed for stride length *H*; higher BMI significantly predicted lower *H* in older adults (left: β = −0.293, *p* = 0.002; right: β = −0.231, *p* = 0.013) but not in young and middle-aged groups.

**Conclusion:**

These findings suggest BMI affects the temporal structure of gait variability is age-dependent, which may have implications for gait assessment in ageing populations.

## Introduction

To meet the demands of daily life, gait adapts continuously to external factors, such as terrain (Gates et al. 2012; Voloshina et al. 2013), and internal factors, such as body mass (Capodaglio et al. 2021; Choi et al. 2021; da Silva-Hamu et al. 2013; Popescu et al. 2025). Higher body mass increases the load on the lower limbs and challenges the balance control system during gait (Lockhart et al. 2019; Neri et al. 2020). Consequently, higher BMI has been associated with impaired postural control and increased fall risk, particularly in older adults (Lockhart et al. 2019; Neri et al. 2020). Understanding how body mass influences gait is therefore important for identifying factors that may contribute to mobility limitations and falls.

Gait variability, the natural stride-to-stride fluctuations that exist during gait, has also been associated with mobility limitations and falls especially in the elderly (Gabell and Nayak 1984). Gait variability is commonly investigated in two different ways: magnitude, reflecting how much successive strides differ from one another, and temporal structure, reflecting how those fluctuations are organized over time (Stergiou 2020). Importantly, magnitude and temporal structure can change independently, meaning variability can increase in size while remaining organized, or become disorganized without increasing in size (Stergiou 2020). However, studies examining how body mass affects gait variability have primarily focused on magnitude (Adouni et al. 2024; Capodaglio et al. 2021; Lee and Shin 2022). Higher body mass has been associated with greater magnitude in spatiotemporal parameters, such as stride length (the distance between consecutive heel contacts of the same foot) and stride interval (the time between consecutive heel contacts of the same foot) (Lee and Shin 2022). Studies examining joint kinematics have also shown that individuals with higher body mass exhibited altered lower limb joint kinematics, including reduced knee and ankle range of motion during gait (Adouni et al. 2024; Capodaglio et al. 2021). Surprisingly, no study has examined whether body mass affects the temporal structure of stride-to-stride variability. However, the temporal structure of gait variability is thought to reflect the adaptive capacity of the locomotor system (Hausdorff et al. 1997; Stergiou and Decker 2011). Healthy gait shows organized fluctuations in gait variability, indicating that the locomotor system can smoothly coordinate stride-to-stride adjustments. A breakdown of this organization, where fluctuations become more random, suggests reduced coordination of stride-to-stride adjustments (Stergiou and Decker 2011). Such breakdown has been observed in aging and neurological diseases and is associated with reduced adaptability (Hausdorff et al. 1997; Stergiou and Decker 2011). Factors that increase mechanical demands on the locomotor system, such as higher body mass, may similarly challenge this coordination and lead to less organized fluctuations. Therefore, the study of the effects of body mass on the temporal structure of gait variability could provide important insight into how gait changes due to increased mechanical demands.

Body mass typically changes across the adult lifespan, and these changes may interact with age-related declines in neuromuscular function to affect gait (Alley et al. 2008; Kim et al. 2022; Lee and Shin 2022). Higher body mass increases mechanical demands on the locomotor system, requiring adaptations to support body weight and control movement during walking (Capodaglio et al. 2021; Lee and Shin 2022). As individuals age, however, the capacity to compensate for higher body mass may decline due to reductions in muscle strength and neuromuscular control. Consistent with this, age and obesity have been shown to interactively influence gait spatiotemporal and muscle activity in individuals with obesity, such that higher body fat percentage is associated with shorter stride length in older adults (Lee and Shin 2022; Maktouf et al. 2020). Those studies have also examined gait variability from the magnitude perspective, ignoring any effects on the temporal structure (Lee and Shin 2022; Maktouf et al. 2020). Therefore, to understand how body mass affects gait variability, investigations are needed that examine individuals across different age groups. That approach can clarify whether the effects of body mass on the magnitude and temporal structure of gait variability are consistent across the adult lifespan or whether they differ with age.

The purpose of the current study was to examine the effects of body mass on the magnitude and temporal structure of gait variability in three different age groups: young, middle-aged, and older adults. Specifically, we calculated and analyzed the gait variability of the stride length and the stride interval recorded from participants who walked at a self-selected pace on a 200-meter indoor track. We hypothesized that higher body mass would predict greater magnitude and more random temporal structure of gait variability. We also hypothesized that these effects would be larger in older adults, whose capacity to compensate for increased mechanical demands may be reduced due to age-related declines in neuromuscular function.

## Methods

### Participants and protocol

**Fig. 1:**
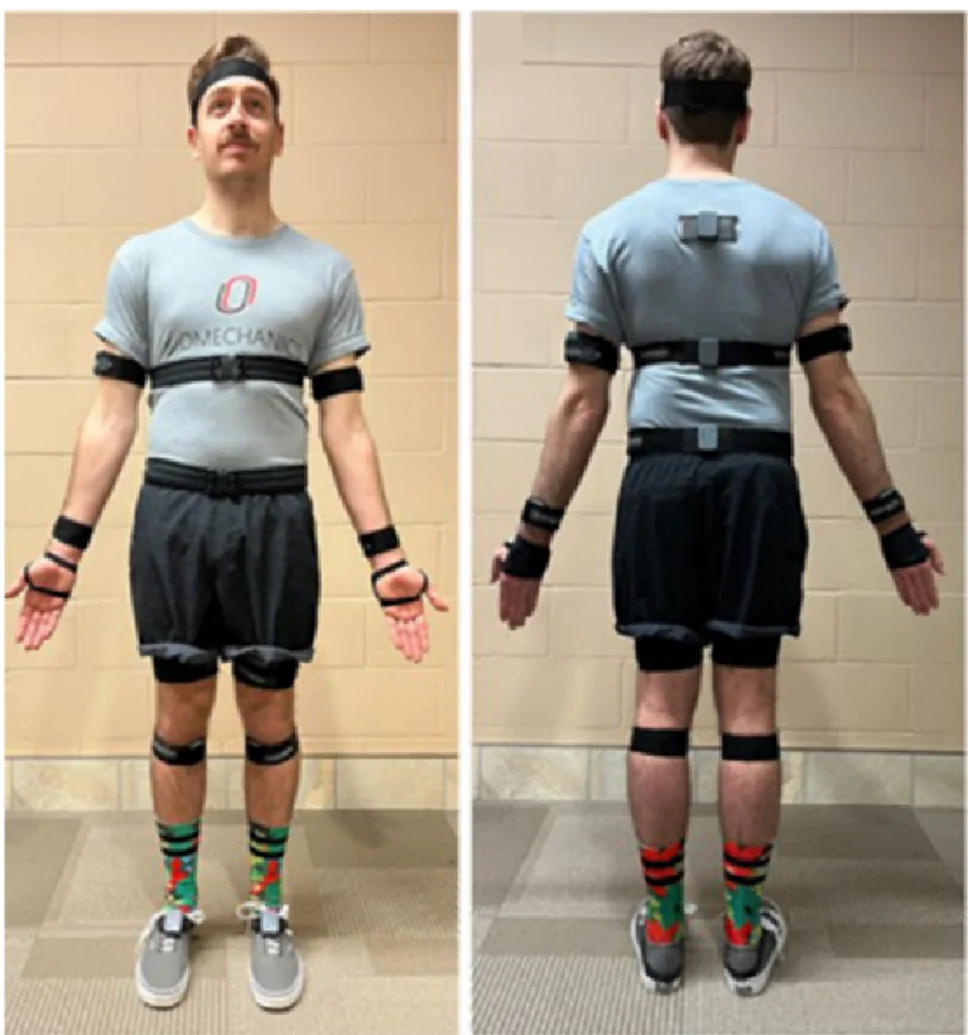
Placement of 16 inertial measurement units (IMUs) on the head, upper trunk, lower trunk, pelvis, upper arms, forearms, hands, thighs, shanks, and feet

We included the gait data from 117 healthy participants (61 women and 56 men) in the NONAN GaitPrint dataset across three age groups (Fig. 1): young adults, middle-aged adults, and older adults (Table 1) (Wiles et al. 2023, 2025a, b). The original data collection protocols received the Institutional Review Board approval. Participants recruitment, sample size justification, and consent form process are detailed in the original dataset publications (Wiles et al. 2023, 2025a, b). The current study involved only secondary analysis of data and did not require additional ethical approval. Participants were free of musculoskeletal, neurological, and lower limb disability. They attended two data collection sessions that were one week apart. Each session consisted of nine 4-minute trials on a 200-meter indoor track, which was conducted at a self-paced speed to reflect participants’ natural gait variability.

**Table 1:** Descriptive statistics of study participants

Age group (age range)	Age (years)	BMI (kg/m²)	Height (m)	Weight (kg)	*N*	Female *N*
Young (19–35)	24.66 (2.79)	23.86 (4.57)	1.74 (0.08)	72.17 (14.85)	32	15
Middle (36–55)	45.77 (5.85)	29.10 (5.39)	1.73 (0.08)	87.83 (18.92)	48	27
Old (over 55)	64.70 (7.65)	27.95 (6.04)	1.71 (0.09)	81.32 (17.01)	37	19

All values are mean (standard deviation)

Data presented as mean (standard deviation). *BMI* body mass index

### Anthropometric measures

Height and weight were measured during the first session. Body mass index (BMI) was calculated as weight (kg) divided by height squared (m²). BMI is a widely used metric in research and clinical settings to characterize body mass relative to height (Nuttall 2015).

### Gait data collection

Gait data were recorded using a Noraxon Ultium Motion system (Noraxon USA Inc., Scottsdale, AZ, USA) with 16 inertial measurement units (IMUs) placed on upper limb, lower limb, trunk, and head segments (Fig. 1) at a sampling rate of 200 Hz. Stride interval was defined as the time between consecutive heel contacts of the same foot. Stride length was defined as the distance between consecutive heel contacts of the same foot. Both parameters were derived from heel contact events detected by the IMUs system. The validity of the Noraxon IMU system has been established in previous studies, which showed that its kinematic output matches that of optical motion capture systems (Berner et al. 2020; Cottam et al. 2022; Park and Yoon 2021). Further details of the data collection and processing procedures are available in the original NONAN GaitPrint dataset descriptors (Wiles et al. 2023, 2025a, b).

### Data processing

Time series of stride intervals and stride lengths were screened and standardized in length to ensure accurate representation of steady-state gait. For consistency, the analysis focused on the final 174 right strides of each trial. Trials with inaccurately recorded or missing heel contacts were excluded. Additionally, trials shorter than the full 4-minute duration were removed to maintain consistency across participants and sessions. In total, 0.912% of trials were excluded.

### Gait variability measures

The magnitude of gait variability was quantified using standard deviation (SD) and coefficient of variation (CoV) for both stride interval and stride length. SD reflects the dispersion of stride values within a trial. CoV was calculated as (SD / mean) × 100, and it provides a mean-normalized measure of variability that facilitates comparisons across individuals and gait parameters.

The temporal structure of gait variability was quantified using the Hurst exponent (*H*). The Hurst exponent characterizes how stride-to-stride fluctuations are correlated over time. *H* ranges from 0 to 1. Persistent fluctuations (*H* > 0.5) occur when a longer stride tends to be followed by another longer stride, and a shorter stride tends to be followed by another shorter stride. Anti-persistent fluctuations *(H* < 0.5) occur when longer and shorter strides tend to alternate. The value of *H* = 0.5 indicates random fluctuations with no temporal correlation between successive strides. In healthy young adults, *H* values for gait parameters typically range from 0.7 to 0.8 which reflects persistent temporal structure (Eke et al. 2002; Ravi et al. 2020). *H* was estimated using the Bayesian Hurst-Kolmogorov (HK) method implemented via a custom MATLAB function (bayesH) from the NONAN toolbox (Hurst 1951; Koutsoytannis 2003). The HK method uses Bayesian inference to estimate the Hurst exponent from the autocorrelation structure of a time series. Two hundred posterior samples were drawn from each time series, and the median of the posterior distribution was used as the point estimate of *H*. This method provides more accurate estimates than traditional approaches such as Detrended Fluctuation Analysis, particularly for shorter time series (Mangalam et al. 2023; Mangalam and Likens 2025), and has demonstrated reliable performance for series lengths in the range used in the present study (174 strides)(Mylonas et al. 2026).

### Statistical analysis

Linear mixed-effects models were used to investigate the effects of BMI, age, and their interaction on the magnitude (SD and CoV) and the temporal structure (H) of stride interval and stride length. BMI and age were standardized using Z-score normalization to facilitate interpretation of effect sizes. Models were built sequentially, beginning with BMI as a fixed effect and participant as a random intercept. Age was then added as a fixed effect, followed by the BMI × age. Model comparisons were performed using likelihood ratio tests, with significant χ² values which indicates improved model fit. When a significant interaction was observed, simple slope analyses were conducted to examine the effect of BMI at different levels of age (mean ± 1 SD). All statistical analyses were conducted in R version 4.3.0 (RStudio version 2023.03.1) using the *lme4* package (Bates et al. 2015) for fitting mixed-effects models and the *lmerTest* package for obtaining p-values. The Type I error rate was set at α = 0.05.

## Results

The effects of BMI and age on the magnitude and temporal structure of stride interval and stride length are presented in Table 2. For magnitude, BMI was a significant positive predictor of SD for both stride interval and stride length bilaterally. Similarly, BMI significantly predicted CoV for stride length on both sides, though only for left stride interval (Table 2). Age and interaction of BMI and age were not significant predictors in any magnitude models. Marginal *R²* values for SD models were 0.047 and 0.051 for stride interval (left and right respectively) and 0.037 and 0.034 for stride length (left and right respectively), while conditional *R²* values were 0.737 and 0.745 for stride interval and 0.423 and 0.424 for stride length. For CoV models, marginal *R²* values were 0.037 and 0.031 for stride interval and 0.074 and 0.070 for stride length, with conditional *R²* values of 0.694 and 0.709 for stride interval and 0.571 and 0.572 for stride length. Based on that information, the fixed effects explained only a limited portion of the overall variance even though BMI was a significant predictor of magnitude of gait variability.

**Table 2:** Effects of BMI and age on SD, CoV, and *H* value of gait variabilities

Measure	Variable	Side	BMI		Age		BMI × Age	
			β (SE)	*p*	β (SE)	*p*	β (SE)	*p*
SD	Stride interval	Left	0.209 (0.073)	0.005*	−0.018 (0.084)	0.835	0.133 (0.089)	0.138
		Right	0.164 (0.072)	0.025*	−0.080 (0.083)	0.343	0.169 (0.088)	0.058
	Stride length	Left	0.172 (0.058)	0.004*	0.086 (0.065)	0.189	0.100 (0.070)	0.156
		Right	0.170 (0.058)	0.004*	0.065 (0.065)	0.318	0.112 (0.070)	0.115
CoV	Stride interval	Left	0.183 (0.072)	0.012*	0.053 (0.082)	0.523	0.109 (0.087)	0.215
		Right	0.137 (0.072)	0.060	−0.014 (0.083)	0.862	0.155 (0.087)	0.078
	Stride length	Left	0.260 (0.064)	< 0.001*	0.079 (0.073)	0.283	0.147 (0.078)	0.062
		Right	0.256 (0.065)	< 0.001*	0.061 (0.073)	0.409	0.152 (0.078)	0.054
H	Stride interval	Left	−0.051 (0.067)	0.448	−0.178 (0.076)	0.021*	−0.069 (0.081)	0.399
		Right	0.023 (0.070)	0.742	−0.098 (0.080)	0.220	−0.071 (0.085)	0.407
	Stride length	Left	−0.132 (0.055)	0.019*	−0.211 (0.062)	< 0.001*	−0.161 (0.067)	0.017*
		Right	−0.081 (0.056)	0.149	−0.202 (0.062)	0.001*	−0.150 (0.067)	0.027*

Note: *β* standardized regression coefficient, *SE*  standard error, *SD * standard deviation, *CoV* coefficient of variation, *H* Hurst exponent, *R²m* marginal R², *R²c* conditional R². *p < 0.05

**Fig. 2:**
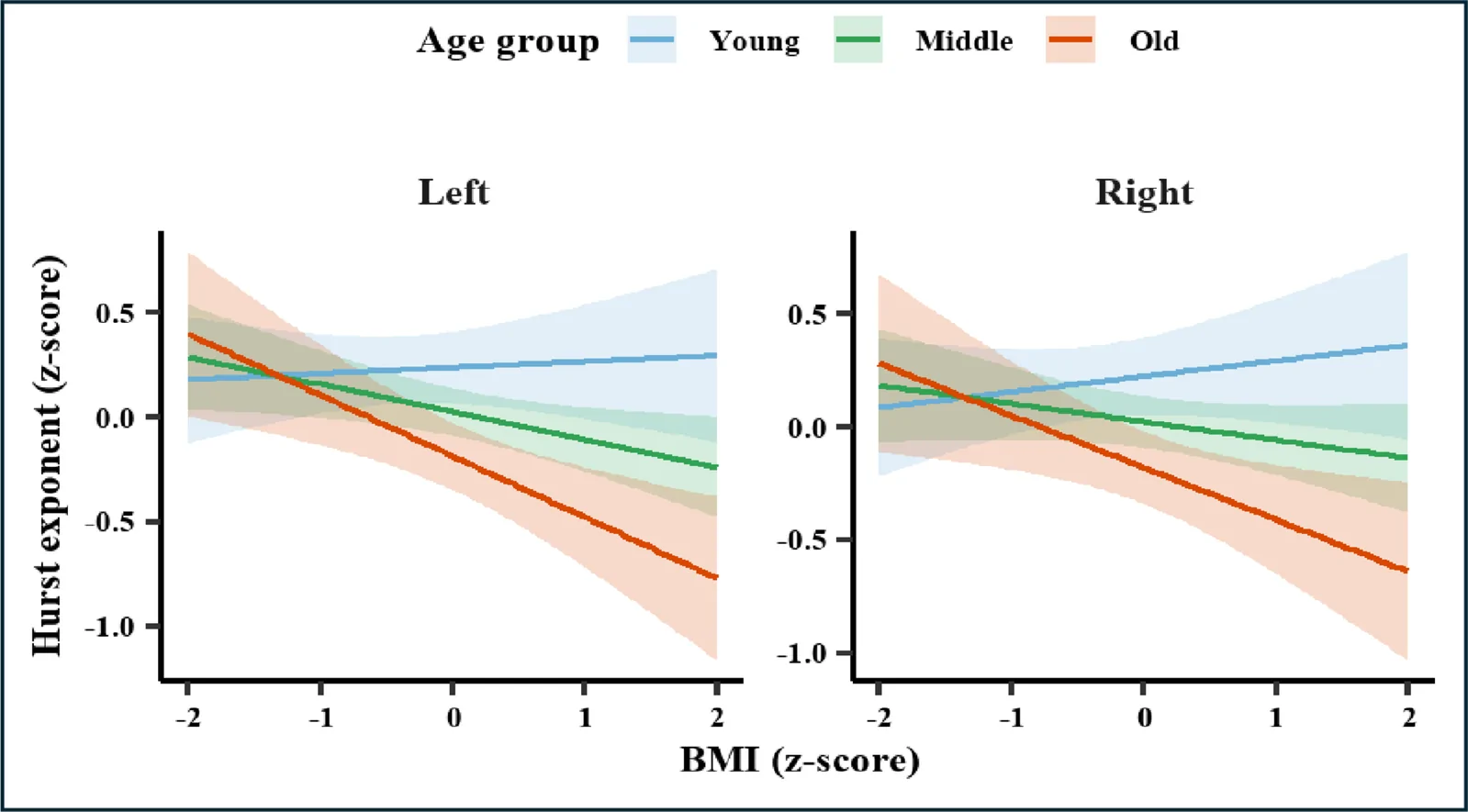
Interaction between BMI and age on the temporal structure of stride length variability. Predicted relationships between body mass index (BMI) and the Hurst exponent (*H*) of stride length are shown separately for the left and right sides. The x-axis represents standardized BMI (z-scores), and the y-axis represents standardized *H* (z-scores). Solid lines indicate predicted values from linear mixed-effects models, and shaded bands represent 95% confidence intervals. Colors represent three age levels: young (− 1 SD), middle (mean), and old (+ 1 SD). The BMI and age interaction was significant for both left (β = −0.161, *p* = 0.017) and right (β = −0.150, *p* = 0.027) stride length *H*. Simple slope analyses revealed that higher BMI significantly predicted lower H in older adults (left: β = −0.293, *p* = 0.002; right: β = −0.231, *p* = 0.013) and in middle-aged adults for the left side only (β = −0.132, *p* = 0.019), whereas BMI was not a significant predictor in young adults (left: β = 0.029, *p* = 0.724; right: β = 0.069, *p* = 0.400)

For temporal structure, BMI alone did not significantly predict *H* for stride interval, however, older age significantly predicted lower *H* for the left side (β = −0.156, *p* = 0.031) but not the right side (β = −0.098, *p* = 0.220). For stride length, the interaction of BMI and age was significant for both sides. Simple slope analyses revealed that the relationship between BMI and stride length *H* differed across age levels: BMI did not significantly predict *H* in young adults (left: β = 0.029, *p* = 0.724; right: β = 0.069, *p* = 0.400), but higher BMI significantly predicted lower *H* in old adults (left: β = −0.293, *p* = 0.002; right: β = −0.231, *p* = 0.013) (Fig. 2). In middle-aged adults, BMI was a significant predictor for *H* in left stride length (β = −0.132, *p* = 0.019) but not right (β = −0.081, *p* = 0.149) (Fig. 2). Marginal R² values for *H* models were 0.031 and 0.009 for stride interval (left and right) and 0.060 and 0.045 for stride length (left and right), with conditional R² values of 0.574 and 0.613 for stride interval and 0.400 and 0.388 for stride length. The low marginal R² values indicate that BMI, age, and their interaction explain a small portion of the variance in *H*, while the larger conditional R² values suggest that subject-level random effects accounted for the most amount of the remaining variance.

## Discussion

We set out to examine the influence of body mass on both the magnitude and temporal structure of gait variability in three different age groups of healthy individuals. We observed that BMI positively predicted SD and CoV of stride interval and stride length, and these relationships were not affected by age. This finding supports our hypothesis that greater body mass is a predictor for increased magnitude of gait variability. In contrast, BMI was not a significant predictor in *H* for stride interval in every age group. For stride length, BMI was not a significant predictor of *H* in young adults, but higher BMI predicted reduced persistence in middle-aged (left side only) and older adults (bilaterally). The age-dependent effect of BMI on temporal structure was further supported by a significant BMI and age interaction for *H* in stride length. In addition to that, we found age as a significant predictor for lower *H* in stride interval (left side) and stride length bilaterally, which indicates reduced temporal persistence with aging. Together, these findings suggest that BMI influences magnitude and temporal structure of gait variability through different mechanisms, magnitude predominantly in a Newtonian fashion but temporal structure in a more neuromuscular manner.

The finding that BMI was a predictor for the increased magnitude of gait variability regardless of age suggests that body mass influences gait variability (SD and CoV) through mechanical demands on the musculoskeletal system rather than central control mechanisms. In other words, we observed a more Newtonian response rather than a neuromuscular response (Bates et al. 2020). Essentially, greater body mass increases the mechanical demands of walking regardless of age, requiring higher force production and joint loading with each stride (Capodaglio et al. 2021; Lee and Shin 2022). These demands result in higher magnitude of gait variability. Previous research has shown that individuals with higher BMI show altered strategies during gait, such as increased muscle coactivation around the ankle (Maktouf et al. 2020), wider step width, and prolonged double support time (Choi et al. 2021; Gonzalez et al. 2020; Kim et al. 2021; Martins et al. 2023). These alterations may introduce greater stride-to-stride fluctuations in gait parameters.

In contrast to variability magnitude, the effects of BMI on temporal structure were age dependent. Age was negatively predicted the *H* of stride length, indicating that older adults exhibited reduced temporal persistence. That is, we observed a more neuromuscular response rather than a Newtonian response (Bates et al. 2020). Age-related declines in neural control of gait are well documented, including grey matter atrophy, white matter integrity loss, and reduced motor cortical function (Seidler et al. 2010; Wilson et al. 2019). If BMI affected variability magnitude through these neural mechanisms, we would expect an age effect or interaction of BMI and age, given that aging is associated with decline in neural function. This result also supports previous literature that demonstrated that aging disrupts the long-range correlations of healthy gait (Wiles et al. 2025a, b).

Furthermore, the significant interaction between BMI and age for stride length *H* suggests that in young adults their neuromuscular system was able to maintain the adaptive, organized pattern of stride-to-stride fluctuations despite the increased mechanical demands imposed by greater body mass. In older adults, however, higher BMI was associated with reduced persistence, suggesting that the combination of age-related neuromuscular decline and increased mechanical loading may exceed the system’s capacity to maintain organized temporal structure. This age-dependent effect of body mass on gait variability is aligned with recent findings. Maktouf et al. (Maktouf et al. 2020) examined SD and the mean of spatiotemporal gait parameters (step length and width, step length), center of pressure velocity, and ankle muscle electromyography across four groups: young non-obese, young obese, older non-obese, and older obese adults. They found that age and obesity each had separate effects on gait and increased ankle muscle coactivation, but their combined effect seemed to involve different neuromuscular mechanisms (Maktouf et al. 2020). Overall, we speculate that younger adults have enough neuromuscular capacity to compensate for the added mechanical load of increased body mass and maintain/preserve the healthy temporal organization of their gait variability, whereas older adults are more vulnerable to the effects of obesity with respect to this aspect of gait variability. This may also explain why obese older adults are more likely to fall while young adults are not regardless of a possible increase in body mass.

One of the limitations in this study is that BMI did not distinguish between fat mass and lean mass. Future studies could include absorptiometry or bioelectrical impedance to investigate the precise contribution of fat versus muscle mass in both magnitude and temporal structure of gait variability changes. Additionally, studies with longitudinal designs are needed to determine whether changes in gait variability precede functional decline or falls in older adults with higher BMI. Finally, extending this work to clinical populations with obesity, neurological, and/or musculoskeletal conditions would help determine the generalizability of these findings, especially for designing rehabilitation programs for those populations.

## Conclusion

This study demonstrates that body mass changes gait variability through different mechanisms depending on magnitude or temporal structure. Higher BMI predicted greater magnitude of gait variability regardless of age. In contrast, the effect of BMI on the temporal structure of gait variability was age-dependent. These findings suggest that the effects of BMI on gait variability may differ across age groups and should be assessed accordingly, evaluating both magnitude and temporal structure, especially when examining gait adaptability and fall risk in older adults with higher BMI.

## Data Availability

The publicly available NONAN GaitPrint database was used in this study: https://doi.org/10.1038/s41597-023-02704-z, https://doi.org/10.1038/s41597-024-04359-w, https://doi.org/10.1038/s41597-024-04359-w.
